# Hepatic safety of repeated treatment with pyronaridine‐artesunate versus artemether–lumefantrine in patients with uncomplicated malaria: a secondary analysis of the WANECAM 1 data from Bobo-Dioulasso, Burkina Faso

**DOI:** 10.1186/s12936-021-03593-6

**Published:** 2021-01-29

**Authors:** Yves Daniel Compaoré, Issaka Zongo, Anyirékun F. Somé, Nouhoun Barry, Frederick Nikiéma, Talato N. Kaboré, Aminata Ouattara, Zachari Kabré, Kadidiatou Wermi, Moussa Zongo, Rakiswende S. Yerbanga, Issaka Sagara, Abdoulaye Djimdé, Jean Bosco Ouédraogo

**Affiliations:** 1 Institut de Recherche en Sciences de la Santé, Direction Régionale de l’Ouest, Bobo Dioulasso, Burkina Faso; 2 Groupe de Recherche Action en Santé, Ouagadougou, Burkina Faso; 3 Ministry of Health, Ouagadougou, Burkina Faso; 4 Malaria Research and Training Center, Department of Epidemiology of Parasitic Diseases, Faculty of Pharmacy, University of Sciences, Techniques and Technologies of Bamako, Bamako, Mali

**Keywords:** Repeated treatment, Pyronaridine‐artesunate, Artemether–lumefantrine, Uncomplicated malaria, Hepatoxicity, Hy’s law, Transaminitis, Hyperbilirubinemia

## Abstract

**Background:**

The use of pyronaridine-artesunate (PA) has been associated with scarce transaminitis in patients. This analysis aimed to evaluate the hepatic safety profile of repeated treatment with PA versus artemether–lumefantrine (AL) in patients with consecutive uncomplicated malaria episodes in Bobo-Dioulasso, Burkina Faso.

**Methods:**

This study analysed data from a clinical trial conducted from 2012 to 2015, in which participants with uncomplicated malaria were assigned to either PA or AL arms and followed up to 42 days. Subsequent malaria episodes within a 2-years follow up period were also treated with the same ACT initially allocated. Transaminases (AST/ALT), alkaline phosphatase (ALP), total and direct bilirubin were measured at days 0 (baseline), 3, 7, 28 and on some unscheduled days if required. The proportions of non-clinical hepatic adverse events (AEs) following first and repeated treatments with PA and AL were compared within study arms. The association of these AEs with retreatment in each arm was also determined using a logistic regression model.

**Results:**

A total of 1379 malaria episodes were included in the intention to treat analysis with 60% of all cases occurring in the AL arm. Overall, 179 non-clinical hepatic AEs were recorded in the AL arm versus 145 in the PA arm. Elevated ALT was noted in 3.05% of treated malaria episodes, elevated AST 3.34%, elevated ALP 1.81%, and elevated total and direct bilirubin in 7.90% and 7.40% respectively. Retreated participants were less likely to experience elevated ALT and AST than first episode treated participants in both arms. One case of Hy’s law condition was recorded in a first treated participant of the PA arm. Participants from the retreatment group were 76% and 84% less likely to have elevated ALT and AST, respectively, in the AL arm and 68% less likely to present elevated ALT in the PA arm. In contrast, they were almost 2 times more likely to experience elevated total bilirubin in both arms.

**Conclusions:**

Pyronaridine-artesunate and artemether–lumefantrine showed similar hepatic safety when used repeatedly in participants with uncomplicated malaria. Pyronaridine-artesunate represents therefore a suitable alternative to the current first line anti-malarial drugs in use in endemic areas.

*Trial registration* Pan African Clinical Trials Registry. PACTR201105000286876

## Background

Artemisinin-based combination therapy (ACT) represents the mainstay of recommended treatment for uncomplicated malaria since the early 2000s. According to the World Health Organization (WHO), no alternative to artemisinin derivatives is expected for several years [1]. Currently, the leading artemisinin-based combination is artemether–lumefantrine (AL) [2], and two artemisinin-based combinations were listed as potential replacement of monotherapies with chloroquine or sulfadoxine–pyrimethamine (SP): artesunate–amodiaquine and sulfadoxine–pyrimethamine–artesunate in areas where SP retains good efficacy [2]. These drugs have been extensively evaluated in Africa [3–7] and South East Asia [8–11] for the treatment of single episodes of uncomplicated malaria. Previous studies have consistently reported high efficacy rates on Day 28. Adopted as first-line malaria therapy in most of West African countries, artemether–lumefantrine has been widely and efficiently used in patients with uncomplicated malaria. In comparison to other artemisinin-based combinations, AL has always been associated with either a similar or a lower risk of adverse events in general and particularly regarding elevated alanine aminotransferase (ALT) and aspartate aminotransferase (AST). For these reasons, AL represents an optimal comparator in monitoring the efficacy/effectiveness and safety of developing anti-malarial drugs or new drugs combination therapies [3, 12–14].

In sub-Saharan Africa, where malaria is endemic, several episodes may occur within a year requiring repeated treatment either with the same drug or with different drugs. However, longitudinal data on safety evaluation on patients exposed to repeated treatment with the same artemisinin-based combination are lacking. In addition, following a period of ultimate efficacity of ACT against uncomplicated malaria, parasites with very prolonged elimination time were detected following the use of ACT [15]; since 2007, confirmed resistant strains of parasites to existing ACT were reported [8, 16, 17] highlighting the urgent need to evaluate the efficacy of further artemisinin-based combinations. In line with this urgent need of new effective anti-malarials and to account for the safety of repeated treatment with these drugs on patients, a clinical trial has been initiated to assess the efficacy and safety of two artemisinin-based combinations (pyronaridine–artesunate and dihydroartemisinin-piperaquine) versus 2 comparators (artesunate–amodiaquine and artemether–lumefantrine) in patients with uncomplicated malaria [18–20]. Since efficacy of these 4 artemisinin-based combinations in the context of this clinical trial has already been reported [19, 20], the present work aims to evaluate specifically the hepatic safety of repeated treatment with pyronaridine-artesunate versus artemether–lumefantrine in patients presenting with uncomplicated malaria during the 2-year follow-up period in Bobo-Dioulasso, Burkina Faso.

## Methods

### Study design

This report is a secondary analysis of data from the WANECAM 1 study site of Bobo-Dioulasso, in Burkina Faso. The protocol and standard procedures of this trial were published elsewhere [18–20]. Briefly, the site of Bobo-Dioulasso was part of a multicentre, randomized phase IIIb/IV clinical trial which aimed to compare effectiveness and safety of repeated treatment with pyronaridine–artesunate (PA) or dihydroartemisinin-piperaquine (DHAPQ) versus artemether–lumefantrine (AL) and artesunate–amodiaquine (ASAQ) in participants with uncomplicated malaria during a 2-year follow-up period. On the study site, only AL was used as comparator to PA and DHAPQ. The present work evaluated the site-specific hepatic safety profile of repeated treatment with PA versus AL and factors associated with hepatic adverse events.

### Study period, site and participants

From August 22, 2012 to November 8, 2015 patients of either gender, diagnosed with uncomplicated malaria were enrolled in two primary health facilities, Sakaby and Colsama in the health district of Do (Bobo-Dioulasso, Burkina Faso). Globally, participants were allocated to either PA or AL arms according to a computer-generated randomization list. Subsequent uncomplicated malaria episodes were treated with the same artemisinin-based combination as at enrolment during the follow-up period.

### Sample size

This secondary analysis used the available dataset on the site of Bobo-Dioulasso. According to the study protocol, a total size of 224 participants was needed in the PA arm versus 296 participants in the AL arm to meet the country/site individual size requirement. This sample size was calculated to give sufficient power for an independent country/site data analysis. The full sample size calculation process is described elsewhere [18].

### Treatments and follow up

During each malaria episode, PA and AL were administered orally over three days (days 0, 1, and 2). PA (Shin Poong Pharmaceutical, Ansan, South Korea) was given either as tablets (180 mg of pyronaridine and 60 mg of artesunate) or as granule sachets (60 mg of pyronaridine and 20 mg of artesunate) once daily. AL (Novartis Pharma AG, Basel, Switzerland) was given twice daily as non-dispersible tablets (20 mg of artemether and 120 mg of lumefantrine). All doses were bodyweight-based and directly supervised at the study clinics from day 0 to day2 as described elsewhere [18]. Regular follow up period lasted 42 days with scheduled visits at the clinics on days 3, 7, 14, 21, 28, 35 and 42. Participants were clinically evaluated and any occurrence of an adverse event (AEs) was recorded at each scheduled or unscheduled visit. Early treatment failures were treated with quinine for 7 days while late treatment failures were considered as distinct malaria episode and treated with the same study drug initially allocated (retreatment).

### Laboratory procedures

#### Blood smears and hepatic parameters measurements

Blood films were performed before enrollment and during each subsequent contact with the participants if needed and in accordance to the study procedures [18]. Overall parasitological assessments were performed according to WHO guidelines [21]. In addition, four (4) millilitres of venous blood were drawn into serum clot activator tubes on days 0, 3, 7, 28, and 42 with sterile materiel. Clinical biochemistry tests were performed on participants’ sera using an automated analyzer (ABX Pentra C200® from Horiba Medical, France). Specific hepatic measured parameters were AST and ALT, alkaline phosphatase (ALP), total and direct (conjugated) bilirubin; In case of occurrence of significant adverse event, a weekly monitoring of the specific parameter was initiated until its normalization. Further investigations on hepatitis A, B, C and E viral infections were done if AST and/or ALT were more than 5 times the upper limit of the normal range (> 5 x ULN).

### Outcomes

Hepatic safety outcomes were non-clinical hepatic AEs that occurred during the follow-up. Due to their high sensitivity, hepatic biomarkers have been preferred to clinical signs in defining hepatic AEs. Thus any significant modification in serum concentrations of transaminases (AST/ALT), ALP, total and conjugated (direct) bilirubin since the initiation of the intervention was recorded as an AE according to the definition of the International Council for Harmonization (ICH) [22].

### Statistical analysis

Available data from the site of Bobo-Dioulasso were stored onto a Microsoft Access® database. After extraction and cleaning, data have been analysed using the version SE 15 of the Stata® software. All analyses were done using a modified intention to treat population which included all randomized subjects who received at least one dose of initially allocated study drugs (PA or AL) during first or subsequent malaria episodes. The first episode groups (or first treatment groups) were compared to repeated treatment groups (or subsequent treatment/episodes groups or retreatments) which included all repeated treatments with study drugs. Pearson’s (or Fisher’s) Chi-square test and Student’s t-test was used to compare proportions and means, respectively; p < 0.05 was considered to be statistically significant.

This study also explored the relationship between retreatment with PA and with AL and the study individual hepatic adverse event outcome using logistic regression models. The potential effect of the retreatment with PA and with AL as risk factors of elevated ALT, AST, ALP, direct and total bilirubin was first analysed using univariate logistic regression. Afterwards, significant association between any of the above outcome variables with retreatment with PA and with AL was adjusted for age and gender in a multiple logistic regression analysis. In these models also, a p < 0.05 was considered significant.

## Results

### Study profile

**Fig. 1:**
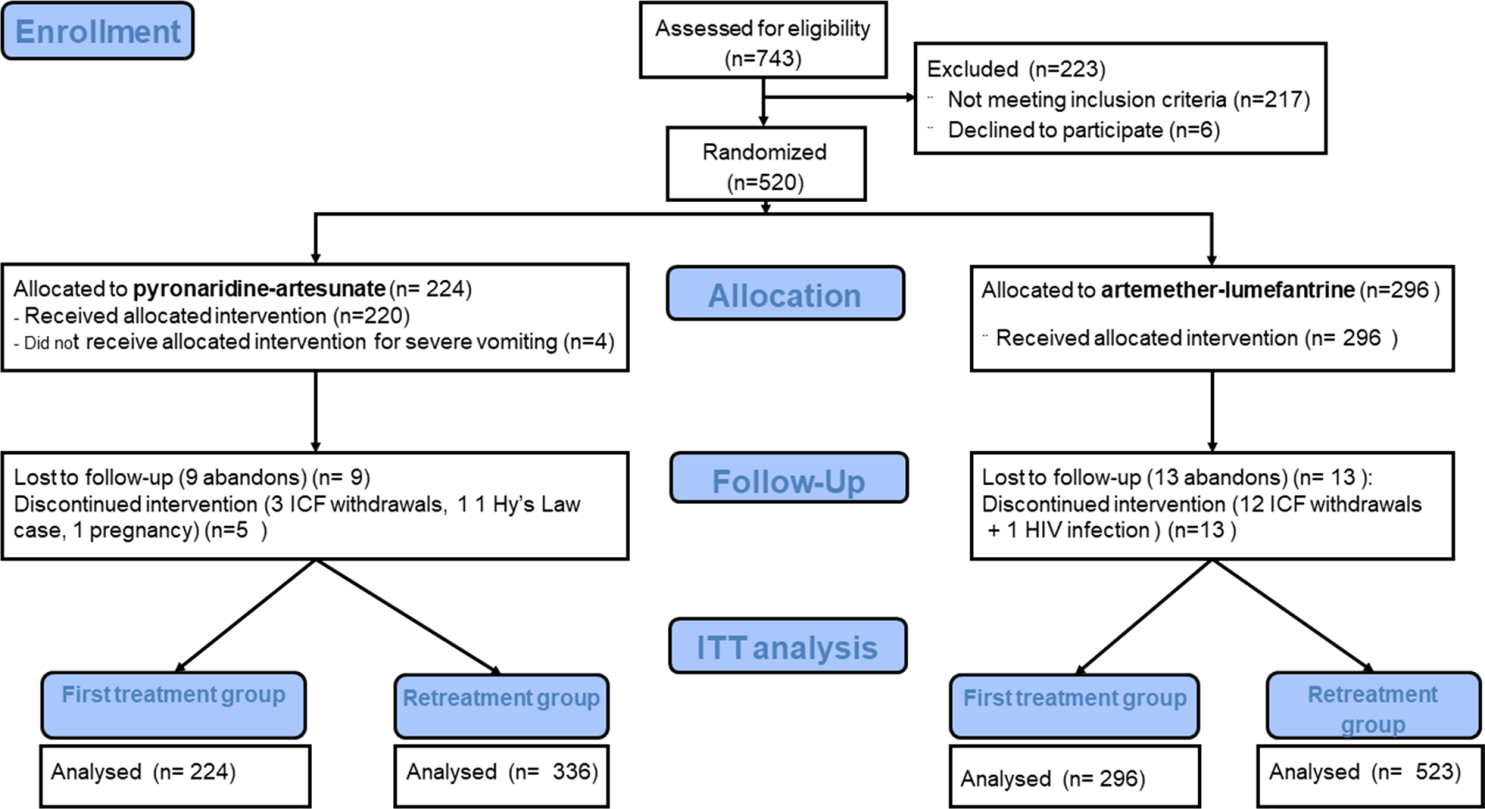
Study Profile. *ICF* Inform consent form; *HIV* human Immunodeficiency virus; *QTc* Corrected QT Interval; *μl* microliter; *AST* Aspartate aminotransferase; *ALT* Alanine aminotransferase; *ITT* Intention to treat

At enrolment, a total of 743 patients were screened of whom 520 were randomized to receive either PA (224) or AL (296). These first malaria episode groups constituted the first treatment groups with study drugs or baseline groups. During the two-year follow-up period, 14 participants in the PA arm and 26 ones in the AL arm were definitely excluded from the study. Reasons leading to these exclusions are given in Fig. 1. In this intention to treat analysis, 859 subsequent malaria episodes were retained over the study period with 336 in the PA arm and 523 in the AL group as shown in Fig. 1.

### Baseline characteristics of the study population

General characteristics of the study population at enrolment are described in Table 1. Mean age of participants was 8.96 ± 6.52 years in the PA arm *versus* 9.29 ± 7.56 years in the AL arm. Participants from the age group 5 to 15 years were predominant in both study arms, accounting for 61.61% in the PA arm *versus* 57.77% in the AL group. Among common presenting symptoms, headache was predominant with 80.36% and 72.97% in the PA and AL arms, respectively. Malaria was mainly due to *Plasmodium falciparum* which caused 99.11% of infections in the PA arm and 98.31% in the AL arm. Globally, in each treatment arm, individual mean values of hepatic parameters and that of haemoglobin concentration at baseline were similar between study groups, as shown in Table 1.

**Table 1:** General characteristics of study population at enrolment according to study arms

Parameters	Pyronaridine-artesunate (PA), N = 224	Artemether–lumefantrine (AL), N = 296
Sex (male), n (%)	95 (42.41)	133 (44.93)
Age, mean ± SD (in year)	8.96 ± 6.52	9.29 ± 7.56
Age groups (in year), n (%)		
≤ 5	58 (25.9)	84 (28.38)
> 5 to ≤ 15	138 (61.61)	171 (57.77)
> 15	28 (12.5)	41 (13.85)
Weight, mean ± SD	25.99 ± 14.46	26.33 ± 14.70
Height, mean ± SD	124.47 ± 24.86	124.76 ± 25.89
Major symptoms, n (%)		
Fever	224 (100)	296 (100)
Headache	180 (80.36)	216 (72.97)
Anorexia	123 (54.91)	135 (45.61)
Vomiting	112 (50.00)	126 (42.57)
Abdominal pain	133 (59.38)	159 (53.72)
Plasmodium species, n (%)		
* P. falciparum*	222 (99.11)	291 (98.31)
* P. malariae*	1 (0.45)	4 (1.35)
* P. ovale*	1 (0.45)	1 (0.34)
* P. falciparum* density, mean ± SD ^§^	16,218 ± 9.50	14,739 ± 9.18
ALT (IU /L), mean ± SD	23.63 ± 11.16	23.61 ± 15.09
AST (IU /L), mean ± SD	29.16 ± 14.83	29.37 ± 15.56
ALP (IU/L), mean ± SD	225.52 ± 95.93	223.98 ± 92.82
Total bilirubin (mg/dL), mean ± SD	1.38 ± 1.06	1.24 ± 0.91
Direct bilirubin (mg/dL), mean ± SD	0.43 ± 0.29	0.41 ± 0.27
Haemoglobin (g/dL), mean ± SD	10.69 ± 1.48	10.49 ± 1.51

SD: Standard deviation; CI: Confidence interval; P: Plasmodium; ALP: Alkaline phosphatase; AST: Aspartate aminotransferase; ALT: Alanine aminotransferase; p: p-value; IU/L: International Units per litre; mg/dl: milligrams per decilitre; g/dl: grams per decilitre; §: geometric mean

### Non‐clinical hepatic adverse events

**Fig. 2:**
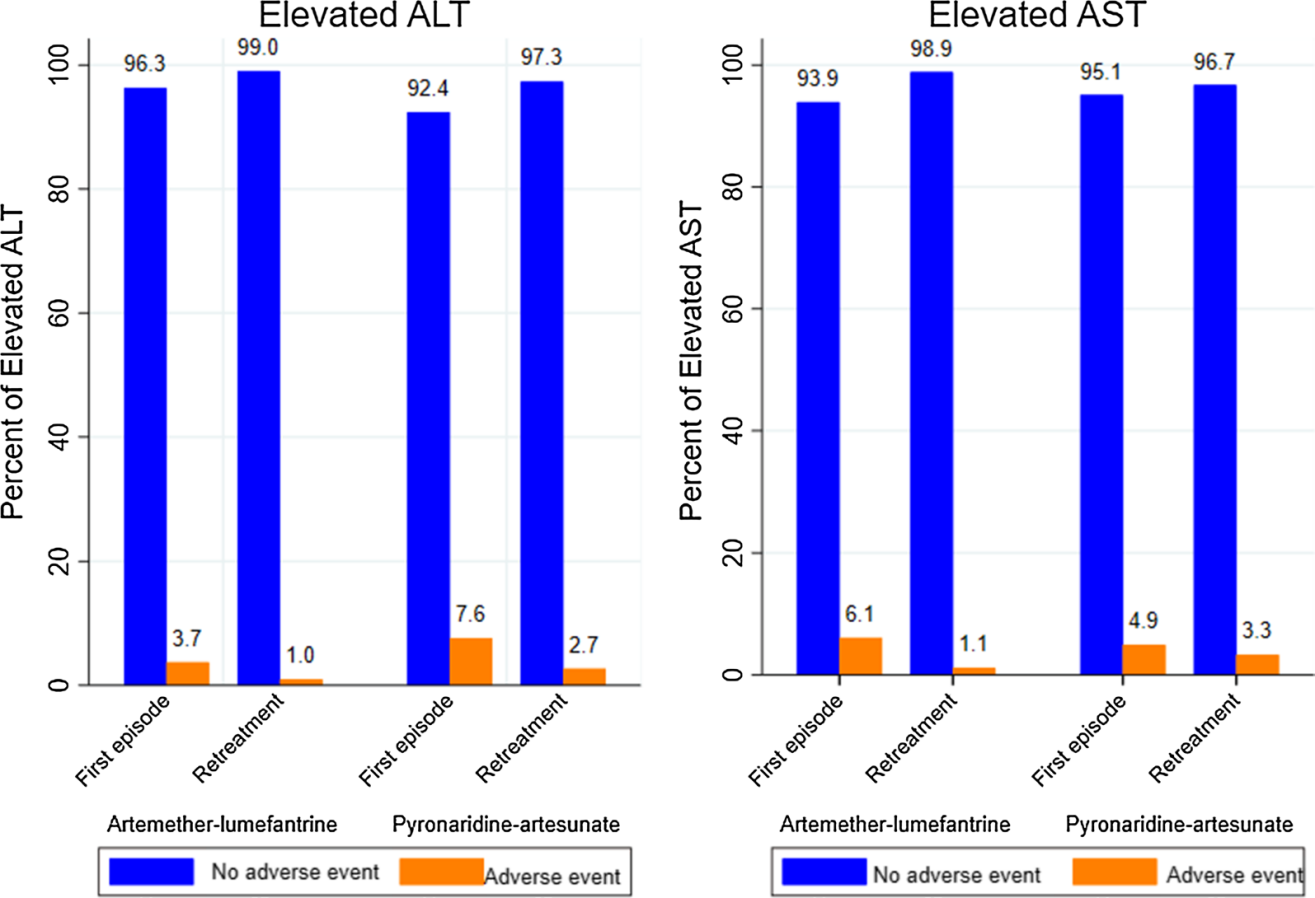
Elevated ALT and AST following first treatment and retreatment with PA versus AL. *AST* Aspartate aminotransferase; *ALT* Alanine aminotransferase. Elevated ALT=ALT > upper limit of the normal range (ULN) for age; Elevated AST= AST > ULN

**Fig. 3:**
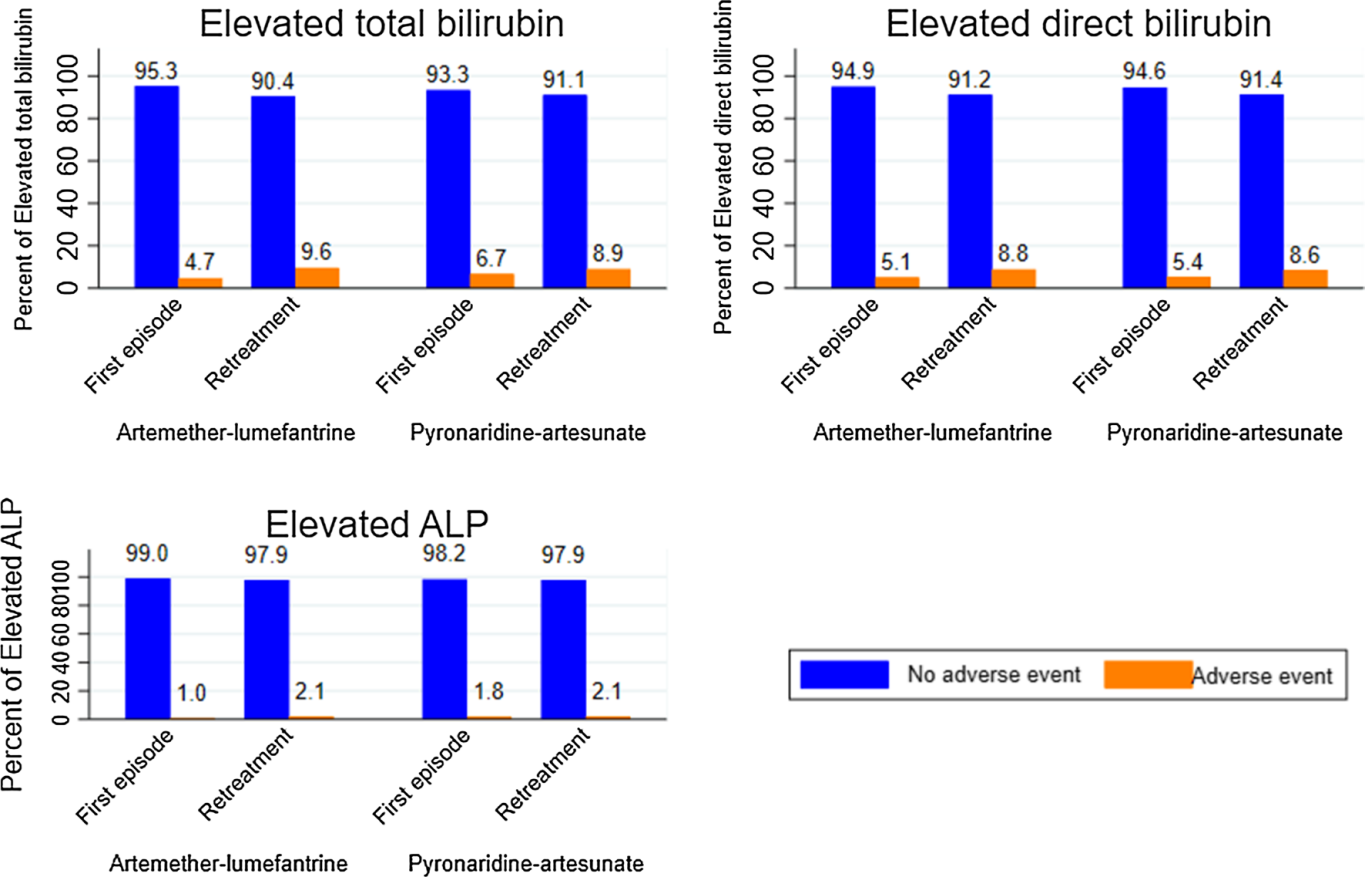
Elevated bilirubin (total and Direct) and ALP following first treatment and retreatment with PA and AL. *ALP* Alkaline phosphatase; Elevated ALP= ALP > ULN; Elevated total bilirubin = total bilirubin > *ULN* Elevated direct bilirubin= direct bilirubin > ULN

At the end of the 2-year follow-up period, from a total of 1379 recorded malaria episodes, 324 hepatic adverse events were registered with a prevalence of 23.5%. Elevated ALT followed 3.05% (40/1379) of all treated malaria episodes, elevated AST 3.34% (46/1379), elevated ALP 1.81% (25/1379), and elevated total and direct bilirubin with 7.90% (109/1379) and 7.40% (102/1379) respectively. More than half (55.25%) of these events (179/324) were recorded in the AL arm. Specific distributions of these adverse events according to the episode groups (first versus subsequent episodes) over study arms are given in Figs. 2 and 3. Proportions of elevated ALT and AST were low in both study arms, globally (Fig. 2). Retreatment groups in AL and PA arms were less likely to experience elevated ALT and elevated AST than the first episode groups; In the AL arm, 0.95%, 95% CI (0.39; 2.27) of elevated ALT were registered during subsequently treated malaria episodes versus 3.71%, 95% CI (2.06;  6.58) during the first treated episodes, p = 0.008. In the PA arm also, less elevated ALT was recorded in the retreatment group [2.67%, 95% CI (1.39; 5.07)] than the first episode group [7.58%, 95% CI (4.76; 11.87)], p = 0.012. In addition, the proportion of elevated AST among subsequently treated participants with AL was lower [1.14%, 95% CI (0.51; 2.53)] than that found among first episode treated participants [6.08% 95% CI (3.86; 9.45)], p < 0.001. In contrast, although the proportion of elevated AST was also lower in the retreatment group of the PA arm [3.27%, 95% CI (1.82; 5.82)] than that of first treatment group [4.91%, 95% CI (2.73; 8.65)], there was no evidence of a significant difference between these proportions, p = 0.377.

In direct comparison of the proportions of elevated ALT among retreated participants in both study arms, there was no evidence of any significant difference between AL (0.95%) versus PA (2.67%) arms, p = 0.058. Similarly, there was a weak evidence of significant difference between the proportions of elevated AST in participants retreated with PA (3.27%) and with AL (1.14%), p = 0.042.

Figure 3 shows a global increased trend of the proportions of elevated total bilirubin, elevated direct bilirubin and elevated ALP in retreatment groups in both study arms comparatively to first episode groups. In the AL arm, the proportion of elevated total bilirubin in the retreatment group was higher [9.56%, 95% CI (7.31; 12.39)] than that of first treatment group [4.72%, 95% CI (2.81; 7.83)], p = 0.014. In the PA arm, the proportions of elevated total bilirubin were similar between first episode group [6.70%, 95% CI (4.07; 10.82)] and subsequent episodes group [8.92%, 95% CI (6.30; 12.49)], p = 0.428.

There was also no evidence of significant difference between the proportions of elevated direct bilirubin in the first treatment groups and the retreatment groups in both AL (5.06%, 95% CI (3.07; 8.24) vs. 8.80% 95% CI (6.64; 11.54), p = 0.053) and PA (5.35% 95% CI (3.06; 9.20) vs. 8.63% 95% CI (6. 05; 12.15), p = 0.145) arms. Similarly, there was also no evidence of significant difference between the proportions of elevated ALP in the first treatment group in the AL arm [1.01%, 95% CI (0.32; 3.09)] and the retreatment group [2.10%, 95% CI (1.16; 3.76)], p = 0.40, as well as in the PA arm between 1.78%, 95% CI (0.67; 4.66) in the first treatment group versus 2.08%, 95% CI (0.99; 4.31) in the retreatment group, p = 1.0.

In direct comparison, proportions of elevated direct bilirubin, elevated total bilirubin and elevated ALP among retreated participants in both study arms were similar. There was no evidence of any significant difference between proportions of elevated direct bilirubin in retreated participants with AL (9.56%) versus PA (8.92%) arms, p = 0.810. The proportions of elevated direct bilirubin in retreated participants in the AL arm (8.80%) was close to that of the PA arm (8.63%), p = 1.00. Similarly, the proportion of elevated ALP after retreatment with AL and that of those retreated with PA was all alike with 2.10% in the PA arm versus 2.08% in the PA arm, p = 1.00.

### Serious hepatic adverse events correlation

**Fig. 4:**
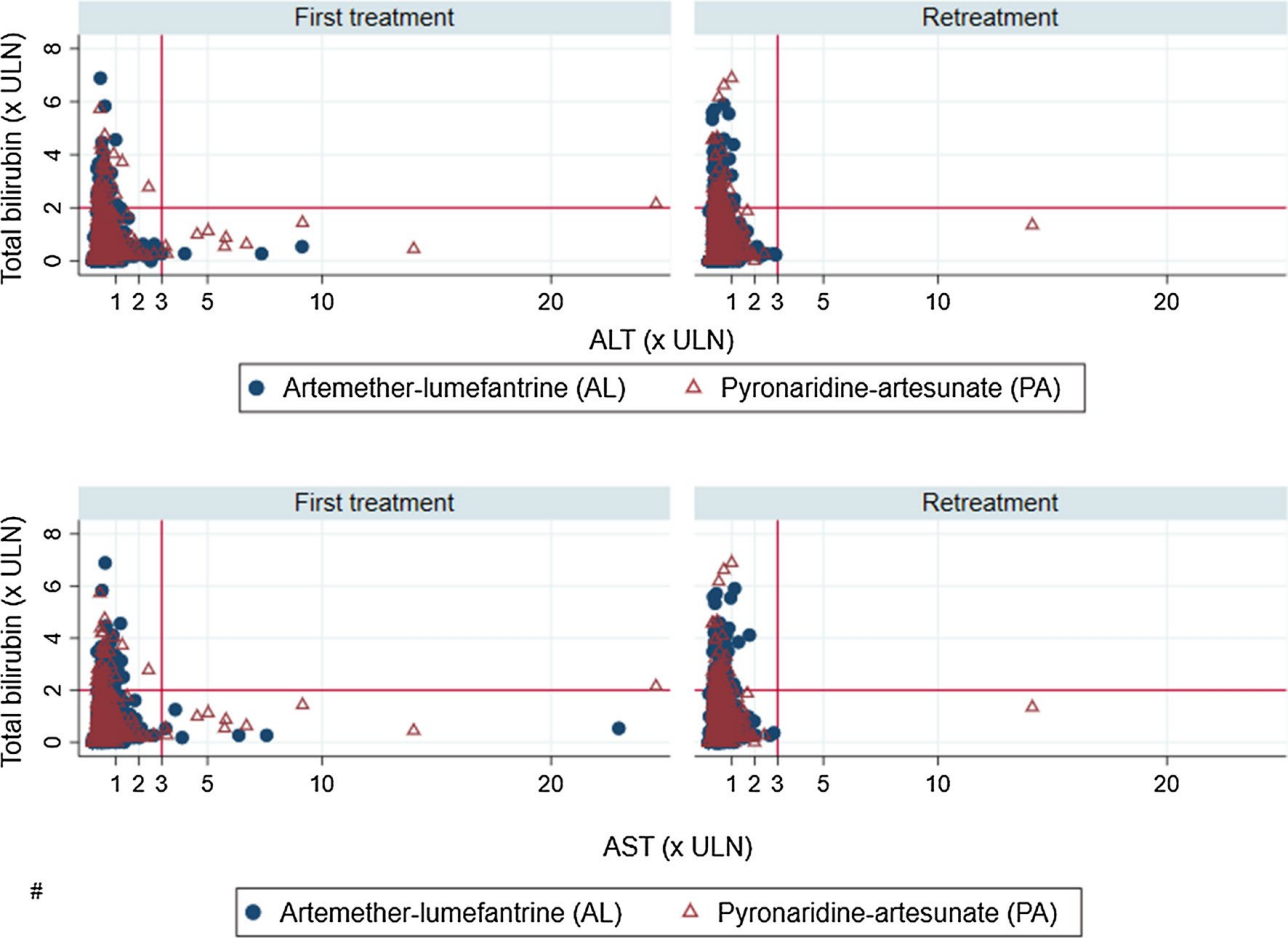
Total bilirubin over ALT and AST by episode groups and treatment arms. The horizontal solid lines represent a total bilirubin level of 2 times the upper limit of normal range (ULN) and the vertical dotted lines correspond to a transaminase level of 3 times the ULN giving a four quadrants distribution of the study participants’ liver enzymes. Participants with isolated hyperbilirubinemia are represented in the top left quandrant and those fulfulling criteria of Hy’s law are in the top right quandrants. Participants with isolated liver injury are spotted in the bottom right quandrants and the bottom left shows study participants whithin the normal range

In Fig. 4, participants’ row total bilirubin was correlated with ALT and AST to identify extreme values and serious hepatic adverse events that fulfilled the Hy’s law criteria in first treatment versus retreatment groups. The top and bottom right quadrants in the retreatment group appear with significant fewer extreme values compared to the first treatment group. Only one case fulfilling Hy’s Law criteria was identified among participants of the first treatment group in the PA arm. This Hy’s law case refers to a three year’s old girl without any clinical manifestation of liver injury. Her liver function parameters (ALT/AST) and the total bilirubin went back to the normal ranges after 21 days. This girl has been mistakenly retreated 4 months later with no more significant hepatic adverse event.

### Retreatment association with non‐clinical hepatic AEs

Table 2 shows the crude association of each non-clinical hepatic adverse events with retreatment with AL and PA. Elevated ALT and elevated AST were 65% and 82% less likely to occur in participants retreated with AL with crude odds ratios (COR) equal to 0.25, 95% CI (0.08; 0.72) for elevated ALT and 0.18, 95% CI (0.07; 0.45) for elevated AST. These participants were also 2 times more likely to have elevated total bilirubin compared to the participants of the first treatment group, COR = 2.13 95% CI (1.15; 3.92). There was no evidence of significant association between retreatment with AL and elevated ALP and elevated direct bilirubin.

**Table 2:** Crude association between hepatic adverse events and episodes groups over treatment arms

Episodes groups		COR (95% CI) with hepatic adverse events				
		Elevated ALT	Elevated AST	Elevated ALP	Elevated direct bilirubin	Elevated total bilirubin
AL	First treatment	1	1	1	1	1
	Retreatment	0.25 (0.08; 0.72) *	0.18 (0.07;0.45) ***	2.09 (0.58; 7.58)	1.80 (0.99; 3.30)	2.13 (1.15; 3.92) *
PA	First treatment	1	1	1	1	1
	Retreatment	0.33 (0.14; 0.76) *	0.65 (0.28; 1.53)	1.17 (0.33; 4.04)	1.66 (0.83; 3.34)	1.36 (0.71; 2.60)

ALT: Alanine aminotransferase; AST: Aspartate aminotransferase; ALP: Alkaline phosphatase; p: p-value; SD: Standard deviation; COR: crude odds ratio; CI: Confidence interval; AL: artemether–lumefantrine; PA: pyronaridine-artesunate

* p < 0.05

** p < 0.01

*** p < 0.001

In the PA arm, participants in the retreatment group were 67% less likely to experience elevated ALT compared to those of the first treatment group, COR = 0.33, 95% CI (0.14; 0.76). Retreatment with PA was not significantly associated with neither elevated AST, elevated ALP nor elevated total and direct bilirubin.

After adjusting for age and gender retreatment with AL was significantly associated with elevated ALT, elevated AST, and elevated total bilirubin. Furthermore, in the AL arm, participants in the retreatment group were 76% and 84% less likely to have elevated ALT [aOR = 0.24, 95% CI (0.08; 0.70)] and elevated AST [aOR = 0.16, 95% CI (0.06; 0.41)], respectively, than those of the first treatment group. Each unit increase in participants’ age was associated with 17% reduced chance to present with elevated AST, aOR = 0.83, 95% CI (0.73;0.93).

In addition, participants in the retreatment group were 2 times more likely to experience elevated total bilirubin, aOR = 2.48, 95% CI (1.31; 4.69). Each unit increase in participants’ age was associated with 5% more chance to present with elevated total bilirubin [aOR = 1.05 95% CI (1.02; 1.09)] as shown in Table 3.

**Table 3:** Adjusted association between hepatic adverse events and episodes groups in the AL arm

Parameters	aOR (95% CI) with hepatic adverse events		
	Elevated ALT LR-p value: 0.025	Elevated AST LR-p: <0.001	Elevated total bilirubin LR-p: 0.001
Episode groups			
First treatment	1	1	1
Retreatment	0.24 (0.08; 0.70) *	0.16 (0.06; 0.41) ***	2.48 (1.31; 4.69) **
Age	0.96 (0.87; 1.05)	0.83 (0.73;0.93) **	1.05 (1.02; 1.09) **
Sex			
Female	1	1	1
Male	1.96 (0.67; 5.77)	0.80 (0.34; 1.84)	1.04 (0.61; 1.75)

AL: artemether–lumefantrine; ALT: Alanine aminotransferase; AST: Aspartate aminotransferase; ALP: Alkaline phosphatase; LR-p: likelihood ratio test’s p-value; aOR: adjusted odds ratio; CI: Confidence interval;

* p < 0.05; ** p < 0.01; *** p < 0.001

After adjustment, retreatment with PA was significantly associated with elevated ALT and total bilirubin. In fact, participants who were retreated with PA were 68% less likely to experience elevated ALT compared to participants of the first treatment group, aOR = 0.32, 95% CI (0.14; 0.74). Repeatedly treated participants of this arm were also 2 times more likely to present with elevated total bilirubin compare to those from the first treatment group, aOR = 2.09, 95% CI (1.01; 4.29). In addition, each unit increase in participants’ age was associated with 12% more chance to present with elevated total bilirubin, aOR was 1.12, 95% CI (1.07; 1.17). However, there was no evidence of significant association of elevated AST with retreatment with PA, aOR = 0.59, 95% CI (0.25; 1.41) as shown in Table 4.

**Table 4:** Adjusted association between hepatic adverse events and episodes groups in the PA arm

Parameters	aOR (95% CI) with hepatic adverse events;		
	Elevated ALT LR-p: 0.041	Elevated AST LR-p: 0.262	Elevated total bilirubin LR-p: <0.001
Episode groups			
First treatment	1	1	1
Retreatment	0.32 (0.14; 0.74) **	0.59 (0.25; 1.41)	2.09 (1.01; 4.29) *
Age	0.98 (0.90; 1.05)	0.93 (0.83; 1.04)	1.12 (1.07; 1.17) ***
Sex			
Female	1	1	1
Male	1.47 (0.64; 3.38)	1.74 (0.69; 4.35)	1.10(0.57; 2.11)

PA: pyronaridine-artesunate; ALT: Alanine aminotransferase; AST: Aspartate aminotransferase; ALP: Alkaline phosphatase; LR-p: likelihood ratio test’s p-value; aOR: adjusted odds ratio; CI: Confidence interval;

* p < 0.05; ** p < 0.01; *** p < 0.001

## Discussion

The efficacy of pyronaridine-artesunate has been largely studied and proven elsewhere [13, 14, 19, 20, 23]. This study aimed to assess the hepatic safety over retreatment of uncomplicated malaria with pyronaridine-artesunate and artemether–lumefantrine and to investigate the relationship between retreatment with study drugs and each identified adverse event. This is a disaggregated data from Bobo-Dioulasso site in Burkina Faso as opposed to the pooled data analysed and published elsewhere [19, 20].

This study shows that the risk of hepatic adverse events occurrence following repeated treatments was similar across study arms. This result argues for the wide and repeated use of pyronaridine-artesunate in any routine health care systems in malaria endemic areas. In fact, artemether–lumefantrine has been intensively studied in clinical trial settings and has shown a satisfactory safety profile. Artemether–lumefantrine has been adopted for several years as a first-line therapy against uncomplicated malaria in many endemic sub-Saharan countries [24–29]. By showing a similar safety profile with artemether–lumefantrine during repeated use in participants, pyronaridine-artesunate can be retained as an alternative to existing first-line anti-malarial drugs in endemic context. Furthermore, the longer plasmatic half-life of pyronaridine-artesunate (14 days) makes this artemisinin-based combination more suitable for extending the delay between two malaria episodes [29, 30].

The results of this study have also shown the particular strength of the association between repeated treatments with pyronaridine-artesunate and artemether–lumefantrine and elevated total bilirubin. Hyperbilirubinaemia following anti-malarial treatment in general, and especially artemisinin-based combinations, is common. In such a context, hyperbilirubinaemia is attributed to the haemolytic process of parasitized red blood cells. This haemolytic process can secondarily be worsened by the ACT-induced haemolysis leading to an unconjugated or indirect hyperbilirubinaemia [31, 32]. Half-life of bilirubin is usually 2–4 h, but can reach 19–21 days when covalently bound to albumin (unconjugated and delta bilirubin). Therefore, hyperbilirubinaemia could persist for several months and could even increase during repeated treatments for subsequent malaria episodes [33, 34].

This study has also shown that repeated treatments with pyronaridine-artesunate and with artemether–lumefantrine did not increase the specific risks of liver injury (with elevated ALT/AST) and of Hy’s law condition. By contrast, repeated treatments with pyronaridine-artesunate and with artemether–lumefantrine trend to reduce these risks with significant lower proportions than those noted after first treatments. This finding could be explained first by the clinical trial setting where participants have been encouraged to visit the health facilities earlier as soon as they feel unwell whatever the symptoms or signs were. In addition, due to the weekly parasitological follow-up procedure until day 42, many late treatment failures have been diagnosed before the onset of clinical symptoms and signs. According to the study protocol, late parasitological or clinical failures identified by day 28 were considered and treated as new episodes. This raises the hypothesis that early diagnosis and treatment of subsequent malaria episodes protected participants from massive hepatocytes and erythrocytes infection and destruction. In contrast, at enrolment participants were similar to those diagnosed in routine settings where delay in healthcare-seeking is common [35, 36]. This suggests that most of participants at enrolment have been seen at study clinics with more advanced illness, high parasite density (in the limit of inclusion criteria) and intensive intrahepatocyte damages. These hepatic injuries that have been triggered by malaria parasites could have been worsened by the use of ACT.

This study has two main limitations. First, the impact of concomitant medications on the liver has not been investigated aside of that of pyronaridine-artesunate and artemether–lumefantrine. In fact, a concomitant drug could independently induced hepatic AEs or interact with study drugs (pyronaridine-artesunate and artemether–lumefantrine) and, therefore, could improve or worsen their impact on the liver [37–39]. Complex algorithms using the Roussel Uclaf Causality Assessment Method (RUCAM) would be needed to adjust for the effect of concomitant drugs [40, 41]. However, as concomitant drugs were prescribed regardless of the treatment arms, their effect can be relativized.

Secondarily, during this study, artemether–lumefantrine was not administered with fatty food to optimize the intestinal absorption of lumefantrine. There was also no daily dietary assessment as recommended in previous studies on artemether–lumefantrine specific efficacy and safety [2, 42, 43]. The lack of concomitant fat intakes with artemether–lumefantrine administration could have reduced its bioavailability and led to a lower global efficacy and to specific risks of hepatic AEs in this arm. Nevertheless, this limitation reflects the reality of the routine clinical practice in malaria endemic areas where patients with uncomplicated malaria mostly present in the health centres with anorexia and are treated immediately with an artemisinin-based combination (including artemether–lumefantrine) before any food intake.

## Conclusions

Pyronaridine-artesunate has a potential as an alternative to artemether–lumefantrine and can help to address the shortage of available, effective and well-tolerated artemisinin-based combinations and to cope with the emergence and spread of anti-malarial drugs resistance. Focusing on the hepatic safety of pyronaridine-artesunate, this analysis of the WANECAM 1 data from the site of Bobo-Dioulasso confirmed that there is no specific increased risk of liver injury with repeated use of pyronaridine-artesunate in participants with uncomplicated malaria. Taking account of its already proven efficacy, the use of pyronaridine-artesunate is, therefore, suitable in malaria endemic countries where patients may experience several episodes mostly during high transmission seasons. In such areas, National Malaria Control Programmes (NMCP) should consider pyronaridine-artesunate as a potential alternative to their actual first-line anti-malarial drugs. However, pharmacovigilance of ACT should be a reality in malaria endemic countries; since artemisinin-based combinations are the most frequently used drugs in these areas, NMCP should undertake effective actions to monitor adopted (first- and second-line) artemisinin-based combinations safety by setting up and effectively running specialized pharmacovigilance centres.

## Data Availability

The datasets used and/or analysed during the current study are available from the corresponding author on reasonable request.

## Abbreviations

PA: Pyronaridine-artesunate
AL: Artemether–lumefantrine
ICF: Inform consent form
HIV: Human Immunodeficiency virus
QTc: Corrected QT Interval
µl: Microlitre
AST: Aspartate aminotransferase
ALT: Alanine aminotransferase
ITT: Intention to treat
SD: Standard deviation
CI: Confidence interval
p: p-value
LR-p: Likelihood ratio test’s p-value
COR: Crude odds ratio
aOR: Adjusted odds ratio
ULN: Upper limit of the normal range
